# Adsorption of azo dye by biomass and immobilized *Yarrowia lipolytica*; equilibrium, kinetic and thermodynamic studies

**DOI:** 10.1007/s11274-024-03949-5

**Published:** 2024-03-22

**Authors:** Amal Hajo Hassan Ibrahim, Nilüfer Cihangir, Neslihan Idil, Y. Doruk Aracagök

**Affiliations:** Department of Biology, Faculty of Science, Hacettepe University, Beytepe-Ankara, 06800 Türkiye

**Keywords:** Adsorption, Adsorption isotherms, Azo dye, Biomass, Immobilization, Kinetics, Thermodynamic

## Abstract

One of the major environmental problems we have today is dye pollution, primarily caused by the textile industry. This pollution has detrimental effects on aquatic life, soil fertility, and human health. Many microbial biosorbents have been documented in the literature for the removal of a wide range of azo dyes commonly employed in the textile industry. However, *Yarrowia lipolytica* NBRC1658 is firstly used as both free and immobilized sorbents for the removal of Reactive yellow 18 (RY18), acid red 18 (AR18) and basic blue 41 (BB41) in this study. The effect of experimental conditions such as pH, biosorbent quantity, dye concentration, contact time, and temperature on dye removal capacity are examined. The research findings demonstrate that the adsorption capacity is higher in biomass compared to immobilized cells. The highest adsorption capacities are observed at pH 2 for RY18 and AR18, while pH 9 is optimal for BB41. Increasing the adsorbent dosage and initial concentration significantly improves the adsorption capacity. The Langmuir model best describes the adsorption process, indicating that the dye attaches to the biosorbent in a single layer, with a uniform biosorbent surface. The removal of the dye occurs through a chemical process on the biosorbent surface, as evidenced by the pseudo-second-order kinetic model. According to thermodynamic analysis, higher temperatures promote greater adsorption of dyes. Our study shows the effectiveness of *Yarrowia lipolyica* NBRC1658 as a biosorbent in the removal of a wide range of industrial dyes.

**Graphical Abstract:**

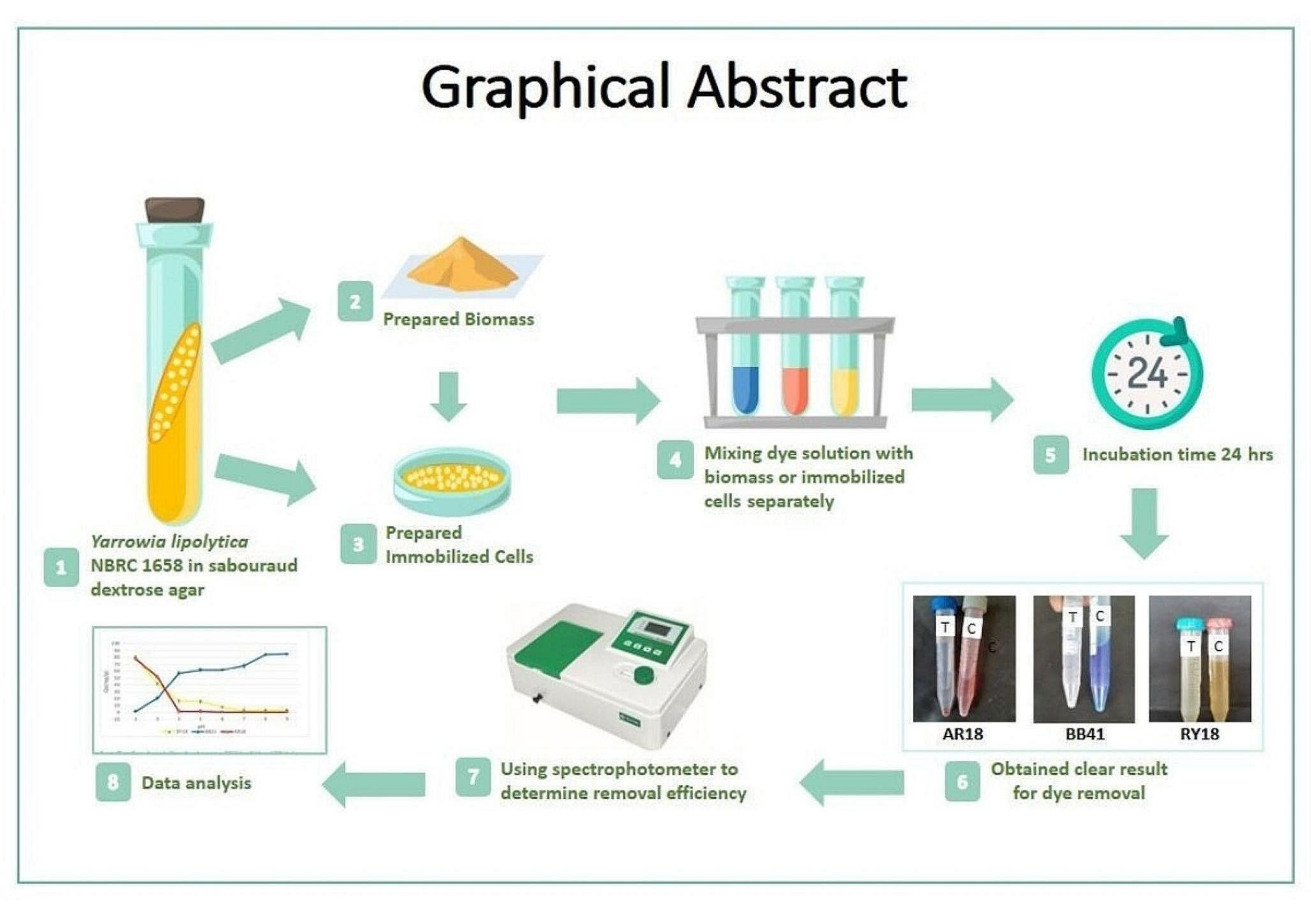

## Introduction

The earliest indications of the use of colour date back to the Stone Age, when cave drawings were discovered (Leidy et al., 2021). All dyes primarily came from organic origins, including lichens, plants, fungi and insects. Since the cystic and commercial dyes were introduced in 1986 the colour industry has undergone a radical transformation. Due to their low cost, they are widely used in many different industrial areas (Solat Rezaei 2014). Currently, more than a hundred thousand synthetic dyes are used industrially and about 7 × 10^7^ tons are produced annually (Karataş 2008; Said Benkhaya et al., 2020).

Based on their uses, dyes are divided into multiple categories. The most popular class of synthetic commercial dyes are azo dyes which are used to colour textiles and make up to 70% of all utilized textile dyestuffs. Unfortunately, the dyeing process contributes significantly to industrial water pollution, with dyestuff operations alone being responsible for up to 20% of this pollution. It is estimated that as much as 84,000 tons of dye are discharged into the environment as a result of these operations (Eleni Routoula &Siddharth and Patwardhan 2020). Therefore, a significant cause of water pollution is thought to be the coloured effluent from the textile sector (M. Starkl et al., 2022).

Moreover, numerous dyes and their active are mutagenic, teratogenic or carcinogenic, presenting significant health risks to living ecosystems (Daneshvar et al., 2005; Siddiqua et al. 2017; Tkaczyk et al., 2020).

Effective approaches for removing colour include chemical, physical, biological, and hybrid treatments (Holkar et al., 2016). Chemo-physical treatments, which include electroflocculation, ozonation, irradiation, membrane filtration, ion exchange and electrochemical destruction are often employed for treating dyes in wastewater (Srinivasan 2010). Despite their effectiveness, chemical and physical treatments have certain drawbacks, including challenges in handling, high costs, and the need for extensive treatment areas. However, biological methods offer several advantages over chemo-physical treatments, particularly in terms of cost and environmental impact. Biological methods are easy to use, safe, and highly effective in removing pollutants. Importantly, they do not result in secondary contamination, making them a more sustainable and desirable approach for addressing environmental pollution (Eleni Routoula et al., 2020). Some families of microorganisms such as bacteria, fungus, actinomycetes, algae and yeast have been recognized for their abilities to neutralise colours (Sharm et al., 2013). One of the most efficient biological methods to remove dyes from an aqueous environment is adsorption which is the process where particles adhere to a material’s surface, including adsorbents like carbon nanotubes, chitin, bamboo-activated carbon, peanut shell and chitosan (Mengelizadeh 2020). Fungal biomasses compared to biosorption agents, exhibit exceptional surface-binding characteristics (Ayele, 2021). Moreover, immobilizing microorganisms have been proposed as a strategy for maintaining effective dyes degradation processes, and many immobilization techniques have been invented to improve wastewater treatment efficiency(Moreno-Garrido 2007). Immobilization procedures involve the interaction of molecules with a polymeric matrix or support, which can come in a variety of shapes and materials, including films, pellets, capsules, and granules (Carvalho et al., 2021). The choice of immobilization technique offers several advantages, depending on the specific application. These advantages include the ability to protect compounds, facilitate catalyst reuse, and simplify product separation processes (de Souza et al., 2022).

Consequently, in this study, the abilities of biomass and immobilized *Yarrowia lipolytica* NBRC1658 to adsorb the azo dyes (Reactive yellow 18 (RY18), acid red 18 (AR18) and basic blue 41 (BB41)) were examined. By investigating the abilities of yeast to remove this range of dyes, the study aims to contribute to a broader understanding of the potential applications of yeast-based adsorption methods in wastewater treatment and pollution control. The novelty of the current study resides in the exploration of yeast’s potential to effectively remove a broader range of dyes that have not been previously investigated. Moreover, the unique aspect lies in the specific utilization of immobilized *Yarrowia lipolytica* NBRC 1658 as the biosorbent. The hypothesis for this study is that both the biomass and immobilized form of *Yarrowia lipolytica* NBRC 1658 will exhibit significant adsorption capabilities for the azo dye’s removal (RY18, AR18, and BB41). Additionally, we aimed to investigate the optimal operational parameters for maximum dye removal efficiency.

## Materials and methods

### The dye

By dissolving 0.1 g of dye in 100 mL distilled water, a stock solution with concentration of 1000 mg/L was prepared. Dilution was used to produce working solutions at the desired concentrations. The characteristics of RY18, AR18 and BB41 are shown in Table 1.

**Table 1:** Characteristics of the RY18, AR18 and BB41 dyes

	Basic blue 41	acid red 18	reactive yellow 18
**CAS No**	12270-13-2	2611-82-7	12226-48-1
**Colour Index**	11,105	16,255	13,245
**molecular weight (g/mole)**	482,57	604.46	906.12
**Class**	Single azo	Single azo	Single azo
**Maximum Wavelength (nm)**	609	508	410
**molecular formula**	C_20_H_26_N_4_O_6_S_2_	C_20_H_11_N_2_Na_3_O_10_S_3_	C_25_H_16_C_l_N_9_Na_4_O_13_S_4_

### The microorganism

The NBRC 1658 strain of *Yarrowia lipolytica*, employed to produce biomass and immobilized cells was cultured on (SDA) sabouraud dextrose agar and kept at 4 °C in the refrigerator for three weeks.

### Chemicals

Commercial RY18, AR18, and BB41 dyes were procured from Alptekin Paint and Chemicals Trade in Turkey. Additionally, various chemicals were obtained from different suppliers: glucose (CAS No. 50-99-7), ammonium sulphate (CAS No. 7783-20-2) and calcium chloride dihydrate (CAS No. 10035-04-8) from Merck in Germany, potassium dihydrogen phosphate (CAS No. 7778-77-0) and sodium alginate (CAS No. 9005-38-3) from Sigma Aldrich in Germany, and magnesium sulphate heptahydrate (CAS No. 10034-99-8) from Isolab Chemicals in Germany. All chemicals were used as received without any additional purification steps.

### Fungal biomass production

*Yarrowia lipolytica* NBRC 1658 was cultivated in the medium prepared using 5 g glucose, 1 g ammonium sulphate, 0.1 g yeast extract, 0.1 g calcium chloride dihydrate, 0.5 g magnesium sulphate heptahydrate and 1 g potassium dihydrogen phosphate dissolved in 1 L of water. *Yarrowia lipolytica* was inoculated into 500 mL of culture medium and incubated at 30 °C for three days (pH 7, 150 rpm). Then, the biomass was filtered from the culture using filter paper and washed three times with distilled water to remove excess culture. The fungal biomass was left to dry for 48 h, then sieved using a 0.15 mm screen, and it was grounded into powder.

### Immobilization of fungal biomass

The obtained biomass was prepared as solution (0.25 g of biomass + 10 mL distilled water) and then mixed with a 1.5% sodium alginate solution (5 mL of biomass solution + 5 mL sodium alginate). The biomass alginate mixture was dropped into 2% calcium chloride solution. The droplets gelled when they encountered Ca (II) cations, generating calcium alginate beads that were left to solidify for 1 h at room temperature before being rinsed twice with distilled water after cross-linking for 24 h at 4 °C. Alginate beads without fungi were prepared as a control in calcium chloride solution and used as a control group in adsorption processes under the same conditions.

### Biosorption studies

Prepared adsorbents (biomass and immobilized *Yarrowia lipolytica* ) were suspended separately in a tube containing 10 mL of dye solution. Tubes with the adsorbent and dye solutions were agitated horizontally (150 rpm) for 24 h. To investigate optimum condition for dye removal, the effect of pH (2–9), initial dye concentration (50- 300 mg/L), adsorbent amount (5–30 mg for biomass, 5-300 mg for immobilized cells), temperature (15–45 °C) and contact time were examined. After the separation of adsorbent using centrifugation, the changes in the concentrations of tested dyes were evaluated using a spectrophotometer (Shimadzu UV-1700).

The adsorption capacity $q$ (mg/g) of tested dyes was determined using the formula:

$$q=\frac{{C}_{i}-{C}_{f}}{w\left(g\right)}\times v\left(L\right)$$

The removal efficiency $r$ (%) was determined using the formula:

$$r=\frac{{C}_{i}-{C}_{f}}{{C}_{i}}\times 100$$

where $q$ = the amount of solute adsorbed from solution, r = % decolorization rate ${C}_{i}$= the initial concentration of the dye, ${C}_{f}$= the final concentration of the dye, $w$ = the weight of the adsorbent (g); $v$ =the volume of the adsorbate (L)(Vanderborght 1977).

### Adsorption isotherms

The adsorption of RY18, AR18, and BB41 on immobilized cells and biomass has been investigated using the Langmuir and Freundlich isotherm models. Adsorption isotherm studies were conducted at 25 ˚C with initial dye concentrations of 50, 100, 150, 200, 250, and 300 ppm. The adsorption studies were performed at pH 2 (for RY18 and AR18) and pH 9 (for BB41).

The Langmuir and Freundlich equations are represented as follows:

Langmuir; $$\frac{1}{{q}_{e}}=\frac{1}{{q}_{m}}+\frac{1}{{q}_{m}{K}_{L}{C}_{e}}$$

Freundlich; $$log{q}_{e}=log{K}_{F}+\frac{1}{nlog{C}_{e}}$$

Where ${q}_{e}$*=* amount of adsorbent adsorbed at equilibrium (mg/g), ${q}_{m}$= maximum capacity (mg/g), ${C}_{e}$= concentration of adsorbate at equilibrium (mg/l), ${K}_{L}$= isotherm constant of Langmuir, ${K}_{F}$= isotherm constant of Freundlich, N = intensity of adsorption (Dada et al., 2012).

Langmuir isotherm features can be described using equilibrium parameters. That is determined by${R}_{L}=\frac{1}{1+{K}_{L}{C}_{e}}$

The isotherm slope can be explained using the value of R in the following ways:

*R* = 1 linear, 0 < *R* < 1 favorable, *R* > 1 unfavorable, *R* = 0 irreversible type of adsorption (McKay 1980).

### Kinetics

Pseudo-first and second-order adsorption kinetics were applied to evaluate the experimental findings. Pseudo-first order was determined by using the Lergergren equation, which expresses as: $\text{ln}\left({q}_{e}-{q}_{t}\right)=\text{ln}\left({q}_{e}\right)-{K}_{1}t$

While pseudo-second order was calculated as: $\frac{t}{{q}_{t}}=\frac{1}{\left({K}_{2}{q}_{e}^{2}\right)}+\frac{t}{{q}_{e}}$(McKay 1980).

### Thermodynamic study

Thermodynamic variables, such as Gibbs free energy (ΔG°,KJ${mol}^{-1}$), entropy (ΔS°, KJ${mol}^{-1}$), and enthalpy (ΔH°, KJ${mol}^{-1}$), were used to explain the way adsorption works. The following formula was used to calculate these thermodynamic parameters:

### $${K}_{L}=\raisebox{1ex}{${q}_{e}$}\!\left/ \!\raisebox{-1ex}{${C}_{e}$}\right.$$, $$G=-RTln{K}_{L}$$, $$log{K}_{L}=\frac{S}{2.303R}-\frac{H}{2.303RT}$$

Where ${q}_{e}$is the amount of adsorbent adsorbed onto the adsorbent (mg/L) at equilibrium condition, ${C}_{e}$ is the solution concentration at equilibrium condition, R is a universal gas constant (8.314 J), T is temperature (k), and ${K}_{L}$is the equilibrium constant. The values of ΔS° and ΔH° are evaluated from the plot of 1/T versus ln${K}_{L}(\text{S}\text{a}\text{r}\text{a}\text{v}\text{a}\text{n}\text{a}\text{n}, 2020)$.

### Statistical analysis

Origin 2023 software was used to describe and analysis isotherms, kinetics models and thermodynamic parameters.

## Results

### Effects of pH

**Fig. 1:**
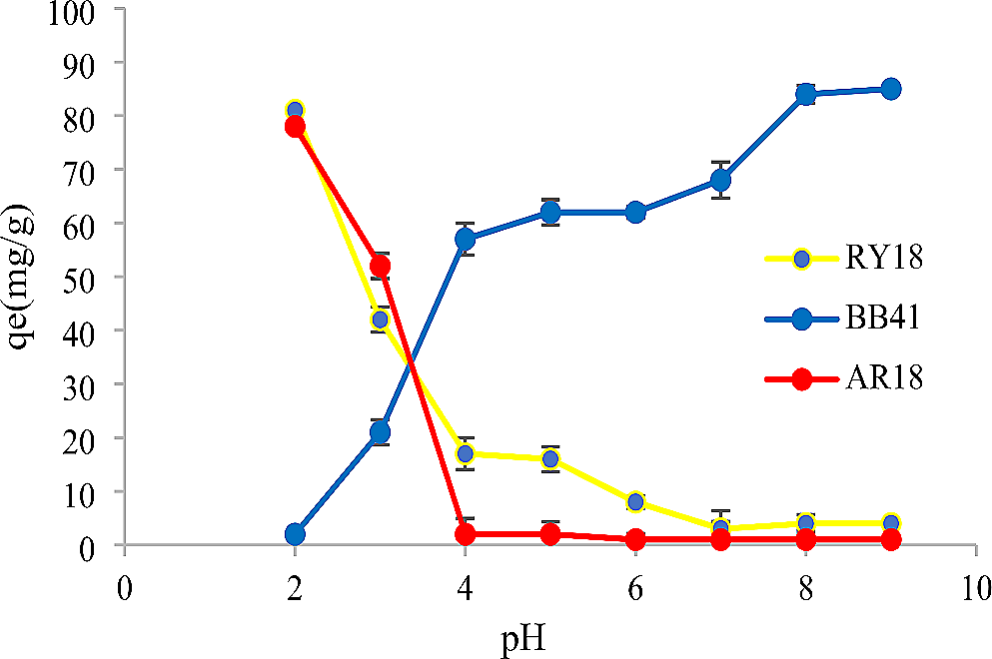
Effect of initial pH on the adsorption of RY18, AR18, and BB41 dyes using biomass (dye concentration: 100 mg/L, volume: 10 mL, amount of biomass: 10 mg, contact time: 24 h, temperature: 25 °C, agitation: 150 rpm)

**Fig. 2:**
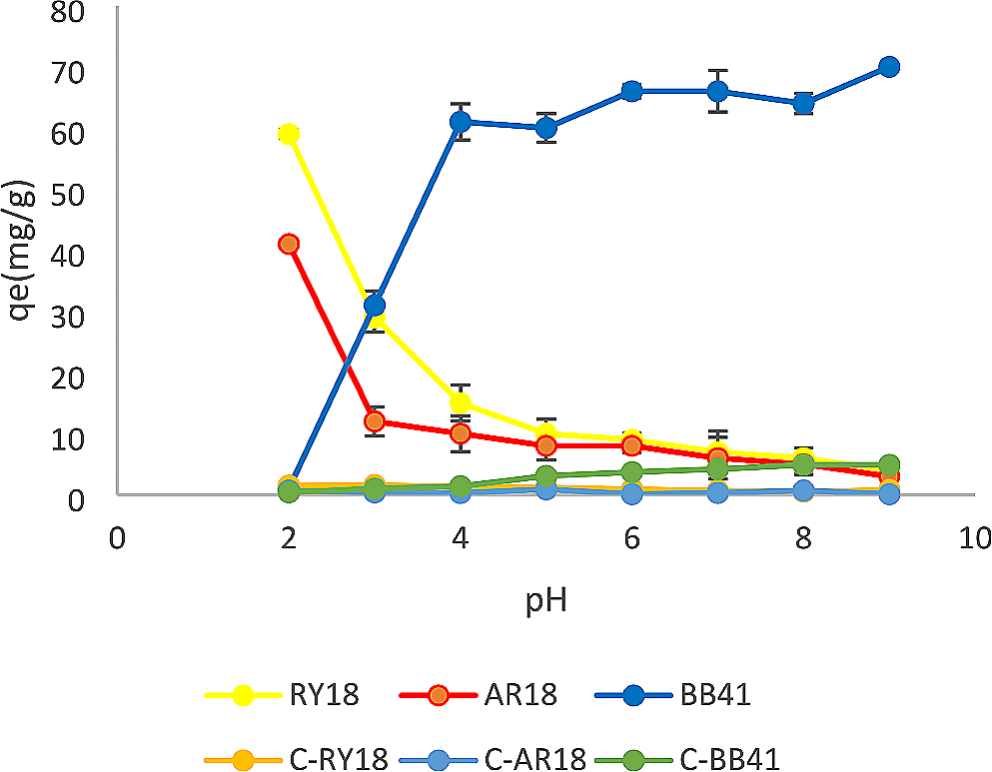
Effect of initial pH on the adsorption of RY18, AR18, and BB41 dyes using immobilized cells and fungus-free alginate beads (C-RY18: Control RY18, C-BB41: Control BB41, C-AR18: Control AR18) (dye concentration: 100 mg/L, volume: 10 mL, amount of immobilized cell: 100 mg, amount of fungus-free alginate beads100 mg, contact time: 24 h, temperature: 25 °C, agitation: 150 rpm)

The process of adsorption was significantly affected by the pH of the dye solution. The pH of the dyes was adjusted using 1 M HCl and 1 M NaOH solutions. Subsequently, a series of experiments were performed, encompassing a pH range from 2 to 9. Based on the data presented in Figs. 1 and 2, it can be observed that the maximum adsorption capacity of RY18 was 81 mg/g and 59 mg/g when utilizing biomass and immobilized cells, respectively. Similarly, for AR18, the maximum adsorption capacity was found to be 78 mg/g and 41 mg/g when using biomass and immobilized cells, respectively. These maximum adsorption capacities were achieved at a pH 2. Thereafter, it decreased as the pH value increased, while BB41 showed the highest adsorption capacity at pH 9. Alginate beads without fungal biomass were used as a control in adsorption processes under the same conditions. As the obtained results demonstrated, there has been no effect of alginate beads alone on the adsorption capacity.

### Effect of dye concentration

**Fig. 3:**
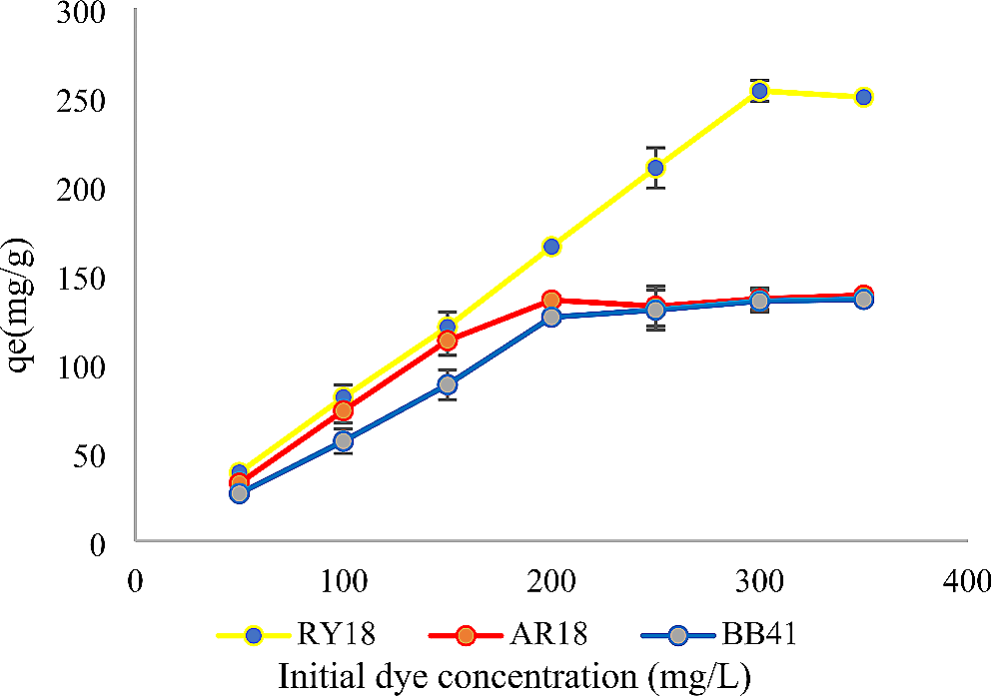
Effect of initial dye concentration on the adsorption of RY18, AR18, and BB41 using biomass (pH: 2 for RY18 and AR18 pH:9 for BB41, amount of biomass: 10 mg, volume: 10 mL, agitation: 150 rpm, temperature: 25 °C, contact time: 24 h)

**Fig. 4:**
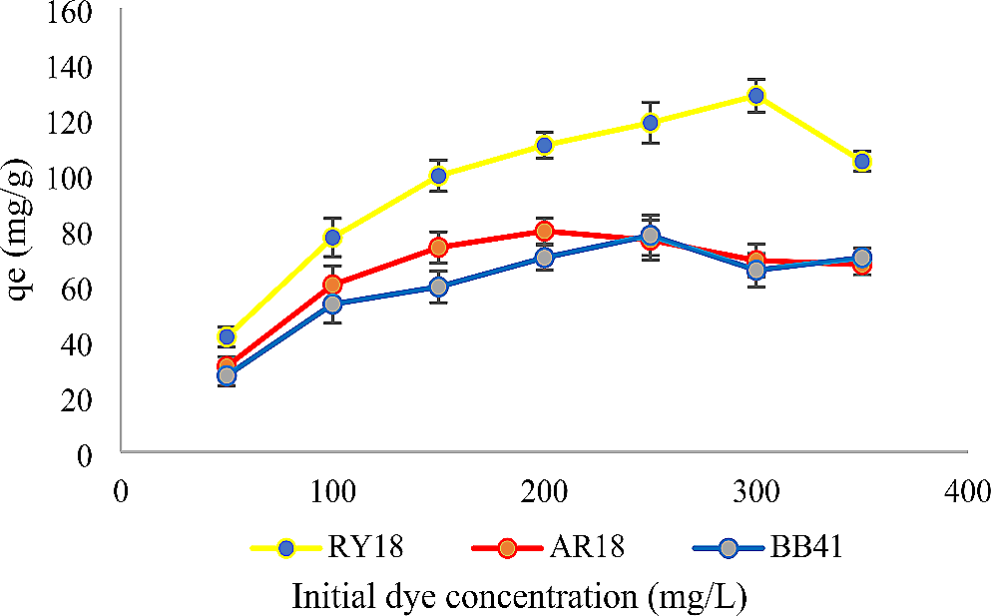
Effect of initial dye concentration on the adsorption of RY18, AR18, and BB41 using immobilized cells (pH: 2 for RY18 and AR18 pH:9 for BB41, amount of immobilized cell:100 mg, volume: 10 mL, agitation: 150 rpm, temperature: 25 °C, contact time: 24 h)

Initially, as indicated by the data presented in Figs. 3 and 4, the decolorization efficiency showed an increasing trend with an increase in dye concentration from 50 to 300 mg/L. However, for RY18, the removal effectiveness started to decline when the dye concentration exceeded 300 mg/L. Similarly, for AR18 and BB41, the removal effectiveness began to decrease when the dye concentration surpassed 200 mg/L. These observations suggest that there is an optimal range of dye concentrations for achieving effective decolorization.

### Effect of biosorbent amount

**Fig. 5:**
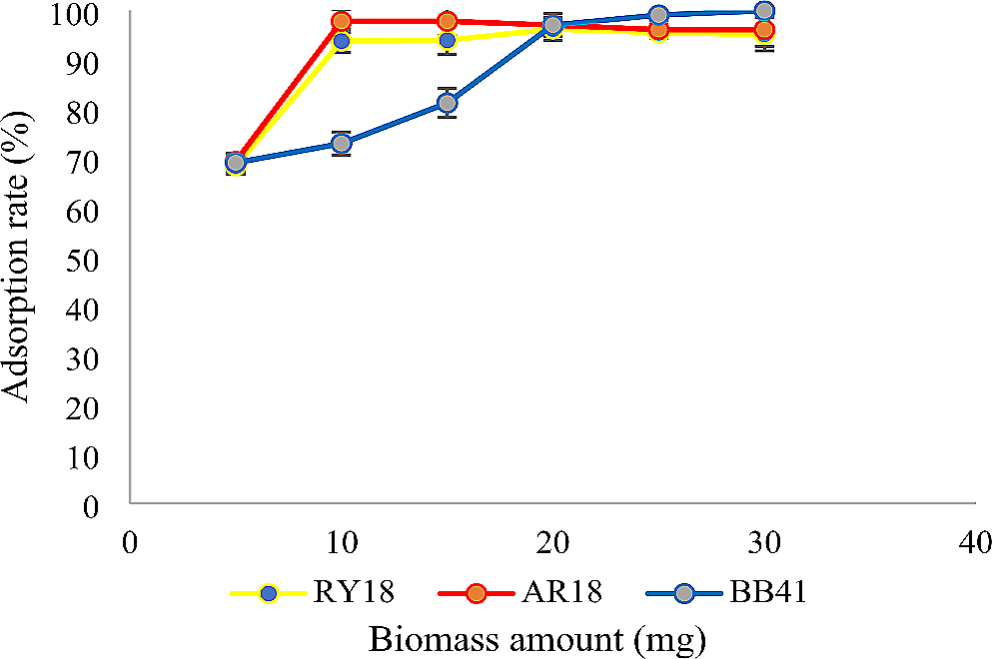
Effect of biomass amount on the adsorption of RY18, AR18, and BB41 (pH:2 for RY18 and AR18 pH:9 for BB41, dye concentration: 300 mg/L, temperature: 25° C, agitation: 150 rpm, contact time: 24 h)

**Fig. 6:**
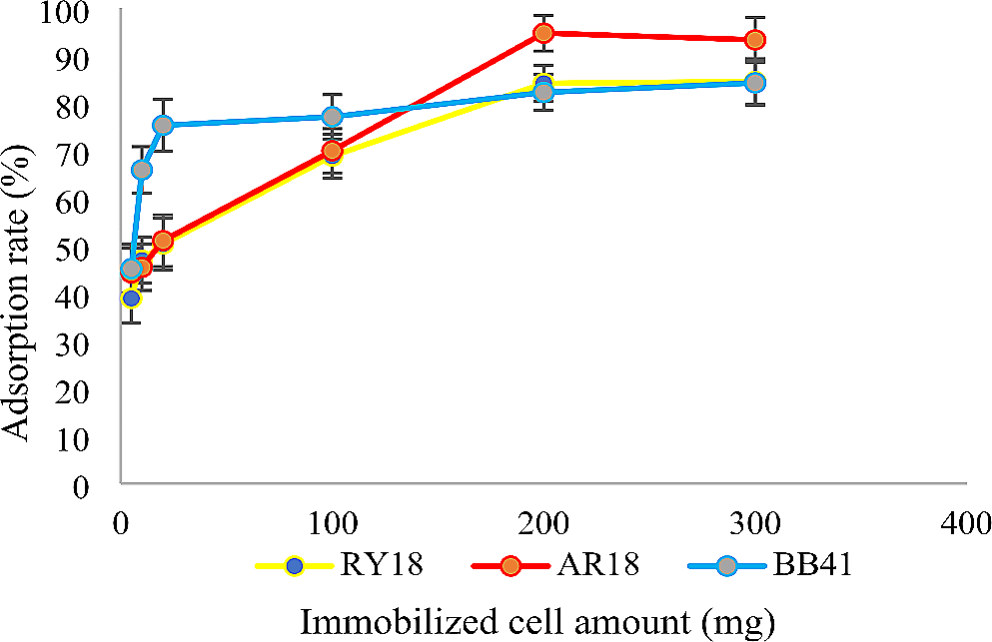
Effect of immobilized cell amount on the adsorption of RY18, AR18, and BB41 (pH:2 for RY18 and AR18 pH:9 for BB41, dye concentration: 300 mg/L, temperature: 25° C, agitation: 150 rpm, contact time: 24 h)

Based on the results, it was observed that increasing the amount of biomass from 5 mg to 10 mg led to an increase in dye removal efficiency. Similarly, increasing the amount of immobilized cells from 5 mg to 300 mg resulted in an increase in dye removal efficiency. However, it was noticed that once the amount of adsorbent was further increased beyond these values, the dye removal rate remained constant. This suggests that there is an optimum amount of adsorbent that maximizes the dye removal efficiency and exceeding that amount does not lead to any significant improvement in the removal rate (Figs. 5 and 6).

### Effect of contact time

The adsorption efficiency of both biomass and immobilized cells for the azo dyes Ry18, AR18, and BB41 was examined within a contact time range of 15–180 min. The results indicate that within the initial 15 min of contact time, both biomass and immobilized cells showed significant adsorption of the azo dyes RY18, AR18, and BB41. When utilizing biomass as an adsorbent, a high percentage of dye removal was observed, with approximately 88% of RY18, 56% of AR18, and 75% of BB41 being adsorbed.

Comparatively, when immobilized cells were used as adsorbents, the adsorption capacity within the first 15 min was slightly lower. Approximately 49% of RY18, 21% of AR18, and 40% of BB41 were adsorbed. This indicates that immobilized cells also have the capability to adsorb the dyes, although to a slightly lesser extent compared to biomass.

### Effect of temperature

The effect of temperature on the percentage of decolorization was examined at four different temperatures (15, 25, 35, and 45 °C). Our study revealed that the adsorption capacity for the removal of RY18, AR18, and BB41 increased with higher temperatures.

When biomass was used as the adsorbent, the adsorption removal rates for RY18, AR18, and BB41 increased as the temperature rose from 15 to 45 °C. Specifically, the removal rates increased from 89 to 94% for RY18, 77–93% for AR18, and 46–99% for BB41. Similarly, when immobilized cells were employed as adsorbents, increasing the temperature from 15 to 45 °C resulted in higher adsorption removal rates for RY18, AR18, and BB41. The removal rates increased from 52 to 93% for RY18, 22–61% for AR18, and 46–83% for BB41.

These findings indicate that higher temperatures positively influence the adsorption capacity of both biomass and immobilized cells, leading to improved removal rates for the tested dyes.

As shown in Tables 2, 3 and 4 the results demonstrate that the adsorption of dye molecules onto biomass and immobilized cells is highly favourable, as indicated by the negative values of Gibbs free energy change (ΔG°,). The positive values of ΔH°,reveal the endothermic nature of the adsorption. Moreover, a positive entropy change (ΔS°,) at elevated temperatures signifies an increase in the degree of freedom of the adsorbed components.

**Table 2:** Thermodynamic parameters for the adsorption of RY18 onto biomass and immobilized *Yarrowia lipolytica*

Temp. (K)	$${K}_{L}$$	ΔG° (KJ$${mol}^{-1}$$)	ΔH° (KJ$${mol}^{-1})$$	ΔS° (KJ$${mol}^{-1})$$	$${R}^{2}$$
Biomass					
288	8,094	-4,8624	21,847	93,018	0,9158
298	11,902	-6,1064			
310	12,584	-6,6952			
318	19,429	-7,8684			
**Immobilized** ***Yarrowia lipolytica***					
288	1,0928	-0,2127	60,27379	208,3544	0,85673
298	1,7738	-1,41998			
310	3,59741	-3,29955			
318	13,687	-6,91756			

**Table 3:** Thermodynamic parameters for the adsorption of AR18 onto biomass and immobilized *Yarrowia lipolytica*

Temp. (K)	$${K}_{L}$$	ΔG° (KJ$${mol}^{-1}$$)	ΔH° (KJ$${mol}^{-1})$$	ΔS° (KJ$${mol}^{-1})$$	$${R}^{2}$$
Biomass					
288	3.431	-2.952	33.07	126.07	0.9022
298	7.638	-5.037			
310	9.465	-5.793			
318	14.038	-6.984			
**Immobilized** ***Yarrowia lipolytica***					
288	1.273	-0.578	41.242	132.63	0.945
298	1.498	-1.002			
310	1.874	-1.608			
318	2.560	-2.560			

**Table 4:** Thermodynamic parameters for the adsorption of BB 41 onto biomass and immobilized Yarrowia lipolytica

Temp. (K)	$${K}_{L}$$	ΔG° (KJ$${mol}^{-1}$$)	ΔH° (KJ$${mol}^{-1})$$	ΔS° (KJ$${mol}^{-1})$$	$${R}^{2}$$
Biomass					
288	0.884	-1.533	80.71	287.13	0.87742
298	2.791	-2.548			
310	26.153	-8.421			
318	32.783	-9.226			
**Immobilized** ***Yarrowia lipolytica***					
288	1.032	-0.077	26.88	92.524	0.83523
298	1.145	-0.345			
310	1.632	-1.253			
318	3.018	-2.920			

### Adsorption isotherms

Analysis of the data presented in Tables 5 and 6 led to a comparison between the Langmuir and Freundlich models. The results demonstrated that the Langmuir model provided a better fit to the experimental data and offered more accurate predictions of the adsorption behaviour in this study.

**Table 5:** Values associated with the Langmuir isotherm:

Dye	Biomass			Immobilized cells		
	${\mathbf{R}}^{2}$	q _max_ (mg/g)	${\mathbf{K}}_{\mathbf{L}}$	${\mathbf{R}}^{2}$	${\mathbf{q}}_{\mathbf{m}\mathbf{a}\mathbf{x}}$ (mg/g)	${\mathbf{K}}_{\mathbf{L}}$
**RY18**	0.985	90.17	0.0209	0.987	187.61	0.0230
**AR18**	0.914	129.53	0.9484	0.960	78.92	0.1052
**BB41**	0.964	729.92	0.0078	0.993	160.25	0.5459

**Table 6:** Values associated with the Freundlich adsorption isotherm:

Dye	Biomass			Immobilized cells		
	${\text{K}}_{\text{F}}$	1/n	${\text{R}}^{2}$	${\text{K}}_{\text{F}}$	1/n	${\text{R}}^{2}$
RY18	7.419	0.676	0.8776	11.688	0.535	0.814
AR18	51.696	0.259	0.8264	20.040	0.289	0.754
BB41	10.495	0.716	0.8481	53.594	0.329	0.823

The superiority of the Langmuir model suggests that the adsorption process follows a monolayer adsorption mechanism, where adsorbate molecules form a single layer on the surface of the adsorbent. The Langmuir model assumes that the adsorption sites on the adsorbent surface are homogeneous and that there is no interaction between the adsorbate molecules (Hashem et al., 2020; Aljeboree et al., 2021).

The calculated R_L_ values from the initial dye concentrations are shown in Table 7. The fact that the R_L_ values fall between 0 and 1 indicates that the adsorption of RY18, AR17, and BB41 on biomass and immobilized cells is generally favourable or effective.

**Table 7:** The R_L_ values according to the initial dye concentrations are as follows:

Concentration (mg/L)	Biomass			İmmobilized cells		
	RY18 ${\text{R}}_{\text{L}}$	AR18 ${\text{R}}_{\text{L}}$	BB41 ${\text{R}}_{\text{L}}$	RY18 ${\text{R}}_{\text{L}}$	AR18 ${\text{R}}_{\text{L}}$	BB41 ${\text{R}}_{\text{L}}$
50	0.4887	0.0206	0.7178	0.4643	0.1597	0.0353
100	0.3233	0.0104	0.5599	0.3023	0.0867	0.0179
150	0.2416	0.0069	0.4589	0.2241	0.0595	0.0120
200	0.1928	0.0052	0.3888	0.1780	0.0453	0.0090
250	0.1604	0.0042	0.3372	0.1477	0.0366	0.0072
300	0.1374	0.0035	0.2978	0.1262	0.0307	0.0060
350	0.1201	0.0030	0.2666	0.1101	0.0264	0.0052

### Kinetics

In this study, the adsorption process was analysed using both the pseudo-first-order model and the pseudo-second-order model. The parameters and correlation coefficients (R2) for these kinetic models are presented in Table 8. The results from the table clearly indicate that the pseudo-second-order model demonstrates the highest correlation with the experimental data, suggesting its superior performance in describing the adsorption process. This finding suggests that the adsorption process in this study follows a chemisorption mechanism. It implies that the adsorbate molecules form strong chemical bonds with the adsorbent surface, leading to a more controlled and stable adsorption process (Venkatesan, 2023).

**Table 8:** The first and second-order adsorption parameters for the removal of RY18, AR18, and BB41 dyes, as well as the experimental and calculated qe values, are as follows:

Dye		pseudo-first-order			pseudo-second-order		
	q_e_, observed (mg/g)	q_e_, calculated (mg/g)	K_1_	R^2^	q_e_, calculated (mg/g)	K_2_	R^2^
Biomass							
RY18	175.4	114.25	-1.538	0.346	176.3	0.00354	0.999
AR18	109.2	74.82	-3.211	0.948	110.13	0.00084	0.986
BB41	142.4	30.93	-3.694	0.821	143.26	0.00323	0.999
Immobilized cells							
RY18	178.7	123.17	-8.51	0.396	176.99	0.00045	0.985
AR18	109.5	63.85	-5.83	0.925	110.13	0.00084	0.986
BB41	109.3	70.37	-4.51	0.898	110.25	0.00071	0.973

Additionally, it can be observed that the calculated q_e_ values are in good agreement with the experimental q_e_ values. This indicates that the pseudo-second-order model exhibits the best correlation with the experimental data.

As a result, while there is a significant difference between the experimental and calculated q_e_ values for the first-order reaction kinetics, this difference is very small for the second-order reaction kinetics.

## Discussion

### Effect of pH

This study aimed to explore the impact of pH levels ranging from 2 to 9 on the adsorption of RY18, AR18, and BB41. Both biomass and immobilized cells were employed in the investigation. Previous studies have reported similar findings. For instance, Chinoune et al. (2016) found that the adsorption capacity of Procion blue HP and Remazol brilliant blue R increased to 43.92 mg/g and 49.23 mg/g, respectively, at a pH of 2 (Chinoune et al., 2016). Additionally, Elumalai et al. (2023) conducted a study on the decolorization of RY 186 and determined that pH 3 was the most effective pH for achieving optimal results (Elumalai et al., 2023). Furthermore, another study focused on the adsorption of methylene blue (MB) revealed that the maximum adsorption capacity was attained at a pH of 9. These results highlight the influence of pH on the adsorption and decolorization processes in different dye systems (Venkatesan et al., 2023).

An explanation of this could be when the pH of the solution increases, the adsorbent’s surface acquires a negative charge, which prevents the dye from adhering to the surface and results in less dye being removed from the aqueous medium (Saravanan et al. 2020b). Moreover, at higher pH values, interchangeable anions are missing from the adsorbent’s outer surface, and subsequently, the adsorption of dye decreases (Jameel et al., 2021).

### Effect of dye concentration

This study focused on investigating the adsorption of RY18, AR18, and BB41 using biomass and immobilized cells as adsorbents, employing various dye concentrations ranging from 50 to 350 mg/L. The maximum efficiency in removing the dye was attained by adhering to the following operational parameters: setting the solution pH to 2 for AY18 and AR18 dyes, pH 9 for BB41. These optimal conditions were consistently maintained throughout the operation, which was conducted at a temperature of 25 °C. Similar findings have been published on the Remazol brilliant blue R adsorption by chemically modified biomass obtained from *Yarrowia lipolytica* which illustrates that the dye adsorption capability was increased as the dye concentration was raised to 150 mg/L (Aracagök 2022).

This observation could be driven by greater utilization of all available functional areas at increasing dye concentrations. Moreover, the removal was improved by increasing the concentration of the dye possibly due to the constant amount of adsorbent, accessibility of adsorbed areas and mass transfer increasing, leading to a rise in adsorption capacity (Nadi et al., 2012). The interaction between dye concentration and available sites on the surface of the adsorbent plays a crucial role in determining the effect of initial dye concentration on dye removal. As the dye concentration increases, the amount of dye adsorbed (mg/g) also increases. However, the proportion of colour removal decreases. This can be attributed to the strong driving force exerted by the initial concentration, which can overcome the mass transfer barrier between the aqueous and solid phases. In other words, the high initial concentration of dye provides sufficient energy to facilitate the adsorption process, resulting in increased dye uptake but reduced efficiency of colour removal (Al-Mahmoud 2020; Cusioli et al., 2020).

### Effect of biosorbent amount

To examine the influence of adsorbent dose on dye removal, varying amounts of biomass (ranging from 5 to 30 mg) and immobilized cells (ranging from 5 to 300 mg) were added to a 10 mL dye solution and mixed at a temperature of 25 °C. The previous study conducted by Aracagök demonstrates that the removal efficiency of RBBR dye increases with an increase in biomass quantity up to a certain limit (Aracagök 2022). In another study, it was found that the efficiency of dye removal increased as the dosage of FeNPs (iron nanoparticles) increased from 0.5 to 2.5 mg. The maximum colour removal was observed at a sorbent dosage of 2.5 mg (Venkatesan et al., 2023). A similar study reported that as the concentration of Fe2 + increased, the decolorization efficiency of Indigo tine also increased significantly (Elumalai et al., 2023). The reason for this is that the increase in biomass quantity leads to an increase in the surface area of the adsorbents and accessibility to additional adsorption sites. The adsorption limit occurs when most of the adsorption sites are saturated due to the high quantity of adsorbent, resulting in a constant removal rate (Ismail Ab Rahman & BS., 2003; Loai, 2018).

### Effect of contact time

In this study, the adsorption efficiency of biomass and immobilized cells for azo dyes RY18, AR18, and BB41 was examined over a contact time range of 15 to 180 min. The study conducted by Ketut et al. in 2020 states that the removal rate increases rapidly within the first 15 min and then steadily increases to reach equilibrium adsorption within 120 min. The maximum adsorption efficiency was observed to be 73% after a contact time of 120 min (Ketut Sudiana 2022). Aracagök’s study also reported a similar finding, with nearly 50% of the dye being adsorbed within the initial 15 min. Similarly, in related study, it was observed that the biosorbent Hordeum murinum rapidly absorbed MB dye during the first few minutes, indicating a strong affinity between the dye and the biosorbent surface. This is due to the existence of several unoccupied adsorption sites on the biosorbent surface. As the contact time increases, there is a gradual increase in adsorption, although at a slower rate because the number of accessible adsorption sites decreases (Al-Mahmoud 2020; Aracagök 2022).

It was observed that biosorption occurs in two stages: In the first stage, dye molecules are rapidly adsorbed due to the abundance of active binding sites on the biosorbent. In the second stage, the biosorption rate slows down, and the removal efficiency decreases (Aracagök 2022). The research conducted by Nor Rahafza and her team revealed that as time progresses during the adsorption process, the available space for adsorption decreases, resulting in a slower rate of adsorption. Their findings provide strong support for the notion that as the adsorbent becomes increasingly saturated with adsorbate, the adsorption process becomes less efficient, leading to a decrease in the overall rate of adsorption (Nagarajan et al., 2022).

### Effect of temperature

Our findings indicate that elevating the temperature promotes the mobility of dye ions, expands the internal structure of the adsorbent, enhances diffusion rates, and facilitates more energetic interactions between the adsorbate and the active sites. These combined effects contribute to an improved adsorption process and the efficient removal of dye molecules (Intidhar et al., 2017). Elif Erkut’s study also supports our findings, demonstrating that as the temperature rises, the adsorption capacity (q) values also increase (Elif 2008).

The thermodynamic parameters presented in Tables 2 and 3, and 4 provide valuable insights into the adsorption process. A negative value of Gibbs free energy (ΔG°) indicates the spontaneity and feasibility of the adsorption process. Additionally, the positive value of enthalpy (ΔH°) confirms the endothermic nature of adsorption. It is generally accepted that physical adsorption involves bonding strengths of less than 84 kJ/mol, while chemisorption can have bonding energies ranging from 84 to 420 kJ/mol. These thermodynamic parameters help to characterize the bonding and energy changes associated with the adsorption process, shedding light on the nature and stability of the adsorbate-adsorbent interactions (Chinoune et al., 2016). A positive value of entropy (ΔS°) indicates a decrease in randomness at the solid solution interface during the adsorption process, specifically during the fixation of the adsorbate on the active site of the adsorbent. This suggests that the adsorption process leads to a more ordered arrangement of molecules at the interface, possibly due to specific interactions between the adsorbate and adsorbent (Begam et al., 2022). This increase in adsorption could be the result of rupturing a couple of inner bonds close to adsorbent surface functional areas, which resulted in an increased fraction of adsorption sites. Moreover, this condition improved the porosity, availability of functional surface areas and membrane density for adsorption (Jameel et al., 2021).

### Adsorption isotherms

Adsorption isotherms are important in the design of adsorption devices, and adsorption equilibrium studies are employed to estimate adsorbent capabilities. The objective of isotherm research is to understand the relationship between pollutant concentration and adsorbent surfaces, hence improving adsorbent utilization for pollutant removal from aqueous solutions. Equilibrium isotherm testing reveals the exchange between adsorbed molecules and the sorbent surface (Venkatesan, 2023).

Among the isotherms investigated, the Langmuir isotherm demonstrates the highest coefficient of association, indicating that adsorption occurs in a single layer. This suggests a well-defined and controlled adsorption process. Additionally, the 1/n Freundlich isotherm constant, ranging from 0 to 1, indicates strong binding between the adsorbate and adsorbent, further supporting the effectiveness of the adsorption process (Venkatesan et al., 2023). Similar results have been published in adsorption of reactive dyes (Orange 16 and Black 5) by three kinds of powdered activated carbons as well as, equilibrium adsorption data of reactive Yellow 145 on magnetic nanoparticles coated with chitosan follows the Langmuir model (Lee & Choi, 2006; Kalkan et al., 2012). On the contrary, isotherm adsorption data of methylene blue dye removal by immobilized *Yarrowia lipolytica*, which fitted onto the Freundlich isotherm model (Mathew Mupa 2018). Whereas equilibrium data of reactive Black 5 adsorption by powdered activated carbon and Afsin-Elbistan fly ash satisfied both Langmuir and Freundlich isotherms models (Eren 2006).

### Kinetics

An analysis of the adsorption process rate provides an understanding of the mechanism of dye molecule removal as it migrates from the solution to the biosorbent surface. This analysis involves fitting the experimental results using two models: the pseudo-first order model and the pseudo-second order model. By employing these models, valuable information can be obtained regarding the kinetics of the adsorption process and the mechanisms involved in the dye removal process (Al-Mahmoud 2020). The pseudo second-order kinetic model has a higher value and linear regression coefficient value compared to the pseudo first-order kinetic model. This suggests that the adsorption process involves chemical adsorption, which may be caused by hydrogen binding between dye and the active functional group in biomass and immobilized *Yarrowia lipolytica* (Xiaoli 2006). A similar result has been recorded in the adsorption of acid dye by acid-activated bentonite (Özcan, 2006). In addition to the study of reactive yellow dye removal from aqueous solution by surface-modified activated carbon (Delonix regia seeds), which revealed a pseudo-second order best-fit model (Saravanan et al. 2020b). Furthermore, the experimental data of reactive Black 5 removal by powdered activated carbon obeyed to a second-order kinetic model. Moreover, this process may involve cationic and anionic forces through electron transfer or exchange between dye and adsorbent (Eren 2006).

## Conclusion

In conclusion, this study emphasizes the significance of biological methods in addressing the prominent environmental issue of dye pollution, primarily associated with the textile industry. The research findings underscore the environmental friendliness and efficacy of utilizing biomass and immobilized *Yarrowia lipolytica* NBRC 1658 as biosorbents for removing a wide range of dyes from aqueous environments. The study investigates several factors, including pH, biosorbent quantity, dye concentration, contact time, and temperature, which affects the adsorption rate.

The results demonstrate that biomass exhibits a higher adsorption capacity compared to immobilized cells. Optimal adsorption capacities are observed at pH 2 for RY18 and AR18, and at pH 9 for BB41. Furthermore, increasing the dosage of the adsorbent and the initial concentration of the dyes significantly enhance the adsorption capacity. The Langmuir model provides the most accurate description of the adsorption process, indicating that the dye molecules attach to the biosorbent in a single layer, forming a uniform surface. The chemical process occurring on the biosorbent surface, as supported by the pseudo second-order kinetic model, is responsible for dye removal. Moreover, the thermodynamic analysis reveals that higher temperatures facilitate increased adsorption of dyes.

For future objectives, it is important to explore the potential for regeneration and reusability of the biosorbent. This investigation would contribute to sustainability and cost-effectiveness by evaluating whether the adsorbent can be effectively regenerated and reused after dye removal. Additionally, scaling up the biosorption process and integrating it with existing wastewater treatment systems should be explored. This assessment will help determine the feasibility and viability of implementing the biosorption technology on a larger scale for various industrial applications.

## Data Availability

No datasets were generated or analysed during the current study.

## Declarations

This research study was conducted from data obtained for laboratory experiment not involving human or animal participants. We consulted with the biotechnology head officer/Hacettepe University who determined that our study did not need ethical approval.
